# Clinical Performance Evaluation of a Rapid Real-Time PCR Assay for Monkeypox Diagnosis: a Retrospective and Comparative Study

**DOI:** 10.1128/spectrum.00510-23

**Published:** 2023-05-16

**Authors:** María Paz Peris, Laura Clusa, Henar Alonso, Cristina Escolar, Blanca Fortuño, Antonio Rezusta, Ana Milagro

**Affiliations:** a Institute for Health Research Aragón, Zaragoza, Spain; b Department of Animal Pathology, Faculty of Veterinary Sciences, University of Zaragoza, Zaragoza, Spain; c Department of Microbiology, Pediatrics, Radiology, and Public Health, Faculty of Medicine, University of Zaragoza, Zaragoza, Spain; d Department of Animal Production and Food Science, Faculty of Veterinary Sciences, University of Zaragoza, Zaragoza, Spain; e Miguel Servet University Hospital, Microbiology, Zaragoza, Spain; Tainan Hospital, Ministry of Health and Welfare

**Keywords:** clinical validation, molecular diagnostics, monkeypox virus, real-time PCR

## Abstract

In an increasingly globalized and interconnected world, the outbreak of an infectious disease in one country can become a worrying health emergency for the whole world. A current example is the 2022 monkeypox virus (mpox) outbreak affecting multiple areas across the world. In this context, strategies to interrupt transmission as soon as possible by identifying cases, clusters, and sources of infection should be developed around the world to prevent these crises. The aim of this retrospective and collaborative study was to perform external clinical validation of the VIASURE monkeypox virus real-time PCR detection kit (CerTest Biotec, Spain) with ready-to-use reagents designed for the rapid detection of mpox. A total of 165 samples with suspected infection were used for this analysis. The standard procedures of the clinical microbiology laboratory of the Miguel Servet University Hospital, using the RealStar *Orthopoxvirus* PCR kit v1.0 (Altona Diagnostics) and bidirectional Sanger sequencing (STAB VIDA, Caparica, Portugal), were considered reference techniques. Furthermore, a subset of 67 mpox-negative samples and 13 mpox-positive samples were routinely tested for clinical diagnosis of other rash/ulcerative pathologies. Accuracy testing resulted in appropriate clinical validation values, as follows: sensitivity, 1 (95% confidence interval [CI], 0.97 to 1); specificity, 1 (95% CI, 0.98 to 1); positive predictive value, 1 (95% CI, 0.93 to 1); negative predictive value, 1 (95% CI, 0.95 to 1). The strength of agreement between assays was almost perfect. The added value is the useful support for the specific diagnosis of mpox infections due to the diagnostic specificity data obtained.

**IMPORTANCE** Given that a large number of mpox outbreaks have been reported worldwide since 2022 in countries in which the disease is not endemic, the main concern for clinicians and global health systems should be to develop effective, available, and easy-to-implement diagnostic strategies to interrupt mpox transmission as soon as possible. This retrospective study demonstrates the satisfactory clinical parameters of a commercially available molecular diagnostic kit for routine testing for mpox in clinical diagnostic laboratories.

## INTRODUCTION

The monkeypox virus (mpox) is a member of the genus *Orthopoxvirus* of the family *Poxviridae*; other members include cowpox virus, vaccinia virus, and variola virus (smallpox) (1). The disease is endemic in parts of central and western Africa. In these areas, mpox is present in wildlife, although sporadic transmission to humans can be appreciated. The infection was first seen in a laboratory monkey in 1958 (hence, the mpox name). Although the exact reservoir in these areas is unknown, a 2010 study stated that several species of forest-dwelling rodents are at risk for *Orthopoxvirus* (including mpox) infection. People living in or near these forested areas may have indirect or low-level exposure, possibly leading to subclinical infection (2).

In 2022, several outbreaks were reported for the first time in countries in which the disease is not endemic. Cases with no epidemiological links to travel or imported mammals arose in Europe and other countries around the globe (3). Human-to-human transmission has been confirmed as a major pathway for this outbreak in multiple areas across the world. Poxvirus infections have no racial predilection, and the incidence rates are equal in males and females. Nevertheless, in the 2022 outbreak, patients were primarily male. A study performed by Thornhill et al. (4) with 528 infections confirmed between 27 April 2022 and 24 June 2022 in 16 countries revealed that 98% of patients were men who had sex with men, 75% were white, and 41% were HIV positive. The median patient age was 38 years.

The current outbreak increased rapidly from May 2022 to August 2022; however, the number of global new cases reported weekly has decreased significantly from September 2022. The number of cases is regularly updated on the official websites of the World Health Organization (3), the European Centre for Disease Prevention and Control (5), and the U.S. Centers for Disease Control and Prevention (6).

The development of effective strategies to interrupt transmission as early as possible by identifying cases, clusters, and sources of infection should be the main objective of the global health systems. Clinicians should be aware of this new situation, which presents a different scenario from those of prior outbreaks (7–9). At present, clinical diagnostic laboratories face a shortage of accessible and easy-to-use tools that can aid in quickly diagnosing mpox infections.

CerTest Biotec (Zaragoza, Spain) has recently elaborated the VIASURE monkeypox virus real-time PCR detection kit (VIASURE assay). Prior to this product’s registry and release to the market, it must be externally evaluated with clinical samples (after nucleic acid extraction) or with DNAs of the pathogens extracted from clinical samples, to confirm that the clinical specificity and sensitivity meet the established requirements.

The aim of this retrospective and collaborative project was to evaluate the clinical accuracy of the VIASURE assay using the standard procedure of the clinical microbiology laboratory (CML) of the Miguel Servet University Hospital (Zaragoza, Spain), with the RealStar *Orthopoxvirus* PCR kit v1.0 from Altona Diagnostics (Altona assay) and Sanger sequencing as reference techniques.

## RESULTS

Between 3 June 2022 and 3 October 2022, a total of 165 samples with clinical (presence of ulcers) or epidemiological (positive contact) suspicion of mpox infection were submitted to the CML. The reference technique (Altona assay) yielded 69/165 samples (42%) that were positive and 96/165 samples (58%) that were negative for mpox infection. Among the samples with positive results, 67/69 (97%) were from men and 2/69 (3%) were from women.

Concerning the anatomical sampling locations from mpox-positive patients, 46/69 swabs (67%) were collected from the genital area and 23/69 (33%) from extragenital areas. The latter areas included the abdomen, mouth, face, rectum, thorax, back, and extremities.

In addition, a subset of 80 DNA extracts (67/80 from mpox-negative samples and 13/80 from mpox-positive samples) were tested for clinical diagnosis of other rash/ulcerative pathologies with the Allplex genital ulcer assay from Seegene, which detects cytomegalovirus (CMV), Haemophilus ducreyi, herpes simplex virus 1 (HSV1), herpes simplex virus 2 (HSV2), lymphogranuloma venereum (LGV), Treponema pallidum, and varicella-zoster virus (VZV). A total of 22/80 extracts (27%) yielded positive results. Of the 67 mpox-negative DNA samples, 4 were positive for HSV1, 2 for HSV2, 7 for VZV, and 3 for T. pallidum, and 51 tested negative for all pathogens. Of the 13 mpox-positive DNA samples, 3/13 (23%) presented coinfection; 2 cases presented coinfection with HSV2, and 1 case presented coinfection with HSV1.

All mpox-positive DNA samples were sequenced by STAB VIDA, Lda (Caparica, Portugal), and all generated nucleotide sequences with suitable sizes. The sequences were compared against the NCBI BLAST database (https://blast.ncbi.nlm.nih.gov/Blast.cgi) (accessed in March 2023), which confirmed the presence of mpox. All sequences had identity of >98% with respect to the sequence with GenBank accession number OQ557957.1 (complete genome), belonging to an mpox isolate originating from the United States.

**FIG 1:**
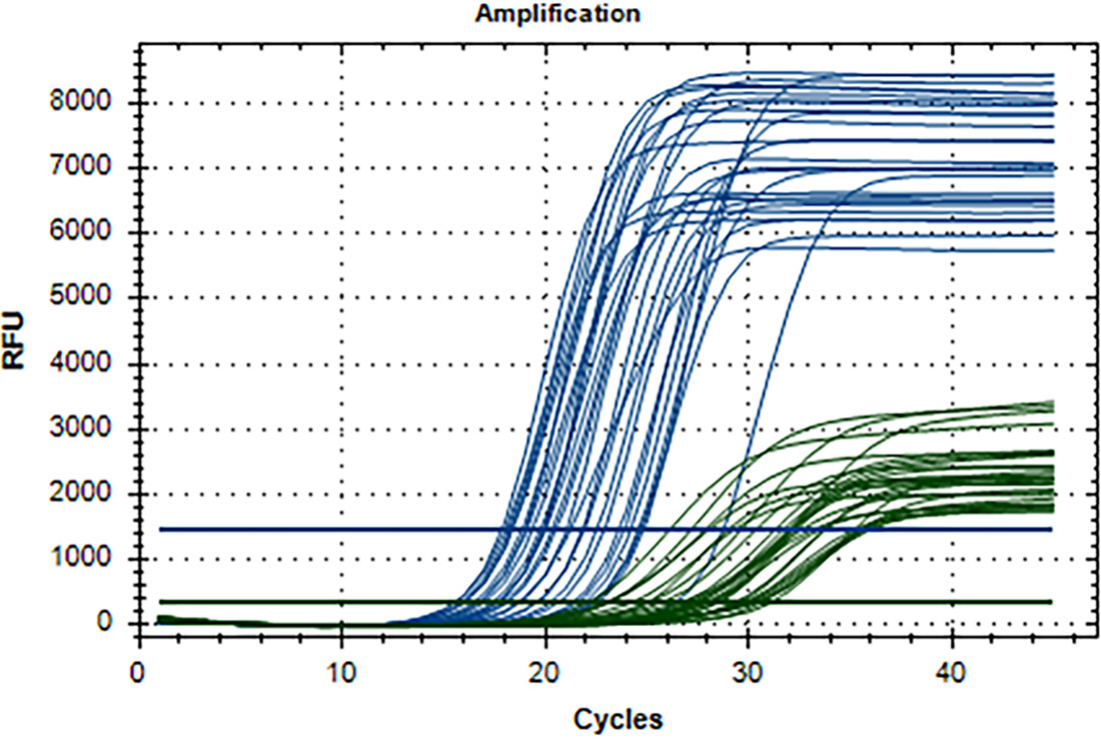
VIASURE monkeypox virus real-time PCR detection kit amplification profiles from mpox-positive and mpox-negative samples, as determined with CFX Manager software. Blue curves correspond to the FAM fluorophore (mpox DNA amplification), and green curves correspond to the HEX fluorophore (β-hemoglobin gene DNA amplification, as an endogenous internal control).

The VIASURE assay correctly identified all of the DNA samples that were positive for mpox. No abnormal amplifications were observed, and all real-time PCR curves from mpox-positive samples presented a sigmoidal shape with the three typical phases, i.e., baseline, logarithmic, and plateau phases (Fig. 1).

The accuracy testing revealed a strong concordance between the CML molecular diagnostic results (Altona assay), the Sanger sequencing results, and the VIASURE assay results, with no discordant results detected (Table 1).

**TABLE 1:** Comparison of the VIASURE monkeypox virus real-time PCR detection kit (VIASURE assay) and the RealStar *Orthopoxvirus* PCR kit v1.0 (Altona assay)

Parameter^*a*^	No. with VIASURE assay result of:		VIASURE validation result
	Positive	Negative	
No. with Altona assay result of:			
Positive	69	0	
Negative	0	96	
Sensitivity (95% CI) (%)			100 (97–100)
Specificity (95% CI) (%)			100 (98–100)
PPV (95% CI) (%)			100 (93–100)
NPV (95% CI) (%)			100 (95–100)
κ			1^*b*^

a PPV, positive predictive value; NPV, negative predictive value.

b The strength of agreement (κ) was almost perfect (κ = 1 [*P* < 0.001]).

Focusing on mpox-positive DNA samples, although threshold cycle (*C_T_*) mean results were observed for the Altona (nonvariola *Orthopoxvirus* species) (*C_T_*, 21.5 [standard deviation [SD], 3.9]) and VIASURE (mpox) (*C_T_*, 21.8 [SD, 4.2]) assays, the *C_T_* values could not be compared because the primers and probes had different targets.

When VIASURE analysis was performed, samples were stored at −20°C for a maximum of 116 days and a minimum of 0 days. For the 10 positive DNA samples that were preserved longest at −20°C (90 to 116 days), the mean *C_T_* values from the Altona assay were 23 (SD, 3.9) for nonvariola *Orthopoxvirus* species and 24.2 (SD, 3.1) for the internal control; the mean *C_T_* values from the VIASURE assay were 22.9 (SD, 4) for mpox and 29.6 (SD, 3.4) for the endogenous internal control. On the other hand, for the 10 DNA samples that were conserved/stored for the shortest period (7 to 22 days), the mean *C_T_* values from the Altona assay were 21.5 (SD, 3.4) for nonvariola *Orthopoxvirus* species and 25.9 (SD, 5.5) for the internal control; the values from the VIASURE assay were 22.1 (SD, 3.9) for mpox and 27.6 (SD, 3.8) for the endogenous internal control. For individual sample information and results, see Table S1 in the supplemental material.

## DISCUSSION

The present study aimed to evaluate the clinical sensitivity and specificity of the VIASURE assay, as well as its accuracy, compared to the reference molecular assay, for mpox infection diagnosis. Our results showed that the VIASURE assay detected all positive samples that had been detected previously with the Altona assay, without any false-positive or false-negative results, even under more unfavorable conditions due to an extra cycle of freezing and thawing. The number of clinical samples used in the study was above the number needed, proving that our results are representative.

Primers and probes from the two real-time PCR assays had different targets; therefore, *C_T_* values cannot be considered equivalent, as Elbaz et al. (10) concluded when they compared two diagnostic kits for mpox detection. It is important to note that the Altona assay does not specifically detect mpox but does detect human variola and nonvariola *Orthopoxvirus* species, as indicated in the methodology. Following the recommendations of the National Centre of Microbiology (Madrid, Spain), the first 15 samples with positive results were sent to their laboratories, where the presence of mpox was confirmed.

The diagnostic accuracy data obtained in this study demonstrated substantial clinical validation values, with sensitivity, specificity, and positive predictive values close to 1. A recent study (10) evaluated two commercial multiplex real-time diagnostic kits, namely, the Novaplex MPXV assay (Seegene) and the Bio-Speedy monkeypox virus assay (Bioeksen), and compared them with a previously described in-house assay (11), demonstrating higher sensitivity values for the commercial kits. According to a CDC laboratory alert from 2 September 2022 (12), mutation at the tumor necrosis factor (TNF) receptor gene might have explained this lower sensitivity of the in-house assays. The mpox target genes in the VIASURE assay were the *G2R* and *F3L* genes, which leads us to consider the fact that no false-negative results were observed in our study. With regard to the Altona assay, the target gene was not disclosed by the manufacturer but, due to the similarity with the results of the VIASURE assay and the Sanger sequencing method, it can be stated as equal.

In terms of demographic data, our study found that most positive samples were from male subjects. Other studies conducted throughout the world during this outbreak concluded that most of the patients affected were middle-aged men who had sex with men (13, 14). The ethical guidelines prevented us from accessing these data in our study.

To date, the disease caused by mpox is manifested mainly with skin/mucosal lesions at multiple sites, including genital, oropharyngeal, or perianal areas (14). An observational cohort study performed in Madrid, Spain, concluded that 76.8% of swab samples were collected from the genital area and 23.2% from extragenital areas (14). Our study showed similar results, with 67% of samples from the genital area and 33% from extragenital areas.

At the current epidemiological stage of the disease, the main public health objectives are to identify cases as quickly and specifically as possible. In the context of detecting mpox in a predominantly at-risk population of individuals who may be coinfected with other organisms or present with an undifferentiated rash disease, an assay with high specificity is also needed. Altogether, infectiousness should also be evaluated in the context of the overall clinical manifestation, including the course of the disease, the lesion location, and the stage (15). Although diagnosis of other rash/ulcerative pathologies was not performed for all samples, our results highlight the importance of syndromic diagnosis; a total of 22/80 samples (27%) yielded positive results for HSV1, HSV2, VZV, and Treponema pallidum. According to the WHO (16), the clinical differential diagnosis that must be considered includes other rash illnesses, such as chickenpox, measles, bacterial skin infections, scabies, syphilis, and medication-associated allergies. A study conducted by Maldonado-Barrueco et al. (17) highlighted the importance of considering other sexually transmitted infections (STIs) in the differential diagnosis of mpox, due to the differences in clinical management of the patient, treatment, and control of outbreaks of the different STIs.

There are two distinct strains of mpox, one originating from West Africa and the other originating from Central Africa. The current outbreak is caused by the West African strain, which is known to be milder (18) than the more virulent Central African strain (19, 20). In an effort to streamline terminology and facilitate communication among virologists and public health experts, the WHO convened a meeting in August 2022 (21). The consensus was to refer to the former Congo Basin clade as clade I and the former West African clade as clade II, with clade II being further divided into two subclades, IIa and IIb. The latter primarily includes the variants causing the 2022 global outbreak (21).

In this sense, the VIASURE assay detects and amplifies a conserved region of *G2R* and *F3L* genes for mpox, covering both the West African and Congo Basin clades (CerTest Biotec, personal communication). Furthermore, the primers used for sequencing were designed to detect a sequence common to both clades, which means that, while the presence of mpox was confirmed, the specific clade could not be identified. However, based on the epidemiological context and other studies conducted at the Carlos III Health Institute (Madrid, Spain) (22), we can assume that all samples correspond to clade IIb.

### Conclusions.

This retrospective study demonstrates the fitting clinical parameters and the strong overall agreement between VIASURE assay results and Altona assay and Sanger sequencing results. The added value observed is the useful support in the specific diagnosis of mpox infections due to the diagnostic accuracy data obtained.

The findings obtained in this study lead to considering the implementation of rapid real-time PCR assays in clinical diagnostic laboratories, allowing high sensibility and specificity values. The high prevalence noted with the studied samples encourages this implementation to avoid treatment failure and stop transmission as soon as possible.

## MATERIALS AND METHODS

### Study design.

This comparative and retrospective study was performed in the CML of the Miguel Servet University Hospital (Zaragoza, Spain) using DNA from a total of 165 samples suspected of mpox. These samples were initially processed by the standard procedure of the CML, which was used as the reference technique. All mpox-positive samples were sequenced by bidirectional Sanger sequencing to confirm the results obtained. Furthermore, a subset of 80 samples (67 mpox-negative samples and 13 mpox-positive samples) were routinely tested for clinical diagnosis of other rash/ulcerative pathologies.

### Ethics statement.

Samples and data from patients included in this study were provided by the Biobank of the Aragon Health System (National Registry of Biobanks B. B.0000873) (PT20/00112), integrated in the Platform ISCIII Biobanks and Biomodels and they were processed following standard operating procedures with the appropriate approval of the Ethics and Scientific Committees. All samples used were anonymized to guarantee the confidentiality of the patients.

The project follows the requirements of Spanish Policy for Biomedical Research 14/2007, of 3 July 2007. The use of all data and samples was approved by the research ethics committee of Aragon (Comité de Ética de la Investigación de la Comunidad Autónoma de Aragón [CEICA]) (project license PI22/412 [date of approval, 5 October 2022]).

### Clinical samples.

A total of 165 samples from 131 men and 34 women with clinical (presence of ulcers) or epidemiological (positive contact) suspicion of mpox infection were used for this study. The locations considered in this study were male and female genital area and extragenital areas (abdomen, mouth, face, rectum, thorax, back, and extremities). DNA extractions and molecular diagnosis were performed during routine testing between 3 June 2022 and 3 October 2022, and VIASURE analyses were conducted retrospectively, between 12 June 2022 and 6 October 2022.

### Test methods.

The automatic DNA extraction system magLEAD 12gC (Precision System Science Co.) was the routine extraction method used in the CML, with a sample volume of 200 μL and a processing time of 19 min. All nucleic acid extractions were eluted in a final volume of 50 μL. Then, mpox diagnosis and, in some cases, clinical diagnoses of other rash/ulcerative pathologies were performed. The DNA leftovers were stored properly at −20°C until used for VIASURE study. Therefore, DNA samples underwent a freeze-thaw cycle.

The routine CML method for mpox detection was the RealStar *Orthopoxvirus* PCR kit v1.0 from Altona Diagnostics (Altona assay). This is designed for the simultaneous detection and differentiation of nonvariola *Orthopoxvirus* species (cowpox virus, mpox, racoonpox virus, camelpox virus, and vaccinia virus) with the 6-carboxyfluorescein (FAM) fluorophore and human variola virus-specific DNA with the Cy5 fluorophore. Additionally, it includes an internal control detected with the hexachlorofluorescein (HEX) fluorophore. A positive-control sample and a negative-control sample provided by the kit were used in each run, and each reaction was performed in a final volume of 30 μL, containing 20 μL of master mix and 10 μL of the DNA template.

For the detection of other rash/ulcerative pathologies, a real-time PCR assay, i.e., Allplex genital ulcer assay from Seegene, was used in the CML for routine diagnosis. This assay can simultaneously detect seven pathogens in one sample, namely, CMV, H. ducreyi, HSV1, HSV2, LGV, Treponema pallidum, and VZV. HSV1 and HSV2 were detected with the FAM fluorophore, the internal control and VZV with Quasar 670 dye, Treponema pallidum and LGV with the CAL fluor red 610 fluorophore, and H. ducreyi and CMV with the HEX fluorophore. A positive-control sample and a negative-control sample provided by the kit were used in each run, and each reaction was performed in a final volume of 20 μL, containing 15 μL of master mix and 5 μL of the DNA template.

The kit under study, the VIASURE assay from CerTest Biotec, detects and amplifies a conserved region of the *G2R* and *F3L* genes for mpox using specific primers and fluorescently labeled probes. Mpox was amplified with the FAM fluorophore and the β-hemoglobin gene (as an endogenous internal control for extraction, amplification, and assessment of sample adequacy) with the HEX fluorophore. The batch used was MPX112L-013 (expiration date, August 2024). A positive-control sample and a negative-control sample provided by the kit were used in each run, and each reaction was performed in a final volume of 20 μL, containing 15 μL of master mix and 5 μL of the DNA template.

All amplifications were carried out in a CFX96 real-time PCR system (Bio-Rad Laboratories, France), and the fluorescence threshold adjustment was automatically performed by the CFX Manager software.

### Bidirectional Sanger sequencing.

Positive samples were sequenced. Briefly, a 327-bp conserved region of the *G2R* and *F3L* genes for mpox was amplified using the following primers: MPX1-Fw (G2R), 5′-GGAAAATGTAAAGACAACGAATACAG-3′; MPX1.3-Seq-Rv, 5′-TCCGTATCCTATTCCACACTTT-3′. The nonpurified PCR product together with the PCR forward and reverse primers (both at 25 pmol) were sent to STAB VIDA, Lda (Caparica, Portugal), for purification of PCR amplicons and bidirectional Sanger sequencing. The sequences obtained were assembled and edited manually by means of CodonCode Aligner software (CodonCode Corp., Dedham, MA). A BLAST search (https://blast.ncbi.nlm.nih.gov/Blast.cgi) was performed using the nucleotide sequences obtained for species classification. Regarding the cutoff value considered suitable for species identification, to our knowledge there is no documented consensus for species definition in virology; a ≥97% cutoff value was adopted from the species definition used in bacteriology (23).

### Data collection and analysis.

The data, including Altona assay, VIASURE assay, and sequencing results, were collected in an Excel file. The Altona assay and sequencing were considered the reference assays to calculate clinical sensitivity, specificity, and negative and positive predictive values (with 95% confidence intervals [CIs]) using Meta-DiSc v1.4 software (24). The strength of agreement (kappa coefficient) between the Altona and VIASURE assay results was determined using SPSS Statistics for Windows v24.0 (IBM Corp., Armonk, NY). The differences were considered significant when the probabilities of equality (*P* values) were ≤0.05. Interpretation of the results on the strength of agreement was performed as described by Schober et al. (25).

The minimum sample size was calculated with WinEpi v2.0 (http://www.winepi.net/winepi2) (26) with the estimate proportion (random sampling and perfect diagnostic) option. The estimated proportion was 0.35%, obtained from the UK population (13). The minimum sample size calculated was 115 individuals, with an accepted error (or precision) of 5% and a confidence level of 99%.
